# Historical Patterns of Diagnosis, Treatments, and Outcome of Epilepsy Associated With Tuberous Sclerosis Complex: Results From TOSCA Registry

**DOI:** 10.3389/fneur.2021.697467

**Published:** 2021-09-08

**Authors:** Rima Nabbout, Elena Belousova, Mirjana P. Benedik, Tom Carter, Vincent Cottin, Paolo Curatolo, Maria Dahlin, Lisa D'Amato, Guillaume Beaure d'Augères, Petrus J. de Vries, José C. Ferreira, Martha Feucht, Carla Fladrowski, Christoph Hertzberg, Sergiusz Jozwiak, John A. Lawson, Alfons Macaya, Ruben Marques, Finbar O'Callaghan, Jiong Qin, Matthias Sauter, Seema Shah, Yukitoshi Takahashi, Renaud Touraine, Sotiris Youroukos, Bernard Zonnenberg, Anna C. Jansen, J. Chris Kingswood

**Affiliations:** ^1^Department of Pediatric Neurology, Reference Centre for Rare Epilepsies, Member of EPICARE Network, Necker Enfants Malades Hospital, Université de Paris, Institut Imagine (Inserm U1163), Paris, France; ^2^Department of Pediatrics, Research and Clinical Institute of Pediatrics, Pirogov Russian National Research Medical University, Moscow, Russia; ^3^Department of Pediatric Neurology, SPS Paediatric Clinic, Ljubljana, Slovenia; ^4^Tuberous Sclerosis Association, Nottingham, United Kingdom; ^5^Department of Respiratory Medicine, Hôpital Louis Pradel, Claude Bernard University Lyon 1, Lyon, France; ^6^Department of Neurology, Tor Vergata University Hospital, Rome, Italy; ^7^Neuropediatric Unit, Karolinska University Hospital, Stockholm, Sweden; ^8^Novartis Farma S.p.A., Origgio, Italy; ^9^Tuberous Sclerosis Association of Bourneville, Gradignan, France; ^10^Division of Child and Adolescent Psychiatry, University of Cape Town, Cape Town, South Africa; ^11^Neurologia Pediátrica, Centro Hospitalar Lisboa Ocidental, Lisbon, Portugal; ^12^Department of Pediatrics and Adolescent Medicine, Medical University of Vienna (Affiliated Partner of the ERN EpiCARE), Vienna, Austria; ^13^Tuberous Sclerosis Association ONLUS, Milan, Italy; ^14^European Tuberous Sclerosis Complex Association, Dattein, Germany; ^15^Zentrum für Sozialpädiatrie und Neuropädiatrie (DBZ), Vivantes Hospital Neukoelln, Berlin, Germany; ^16^Department of Child Neurology, Medical University of Warsaw, Warsaw, Poland; ^17^Department of Neurology and Epileptology, The Children's Memorial Health Institute, Warsaw, Poland; ^18^Department of Neurology, The Tuberous Sclerosis Multidisciplinary Management Clinic, Sydney Children's Hospital, Randwick, NSW, Australia; ^19^Department of Pediatric Neurology, Vall d'Hebron University Hospital, Barcelona, Spain; ^20^Institute of Biomedicine, University of Leon, León, Spain; ^21^Paediatric Neuroscience, Institute of Child Health, University College London, London, United Kingdom; ^22^Department of Pediatrics, Peking University People's Hospital, Beijing, China; ^23^Klinkverbund Allgäu gGmbH Hospital, Kempten, Germany; ^24^Novartis Healthcare Pvt. Ltd., Hyderabad, India; ^25^National Epilepsy Center, National Hospital Organization (NHO), Shizuoka Institute of Epilepsy and Neurological Disorders, Shizuoka, Japan; ^26^Department of Genetics, Centre Hospitalier Universitaire (CHU)-Hôpital Nord, Saint Etienne, France; ^27^First Department of Paediatrics, Athens University Medical School, St. Sophia Children's Hospital, Athens, Greece; ^28^Department of Internal Medicine, University Medical Center, Utrecht, Netherlands; ^29^Pediatric Neurology Unit, Department of Pediatrics, UZ Brussel Vrije Universiteit Brussel (VUB), Brussels, Belgium; ^30^Cardiology Clinical Academic Group, Molecular and Clinical Sciences Research Centre, St Georges University of London, London, United Kingdom

**Keywords:** epilepsy, registry, TOSCA, TSC, tuberous sclerosis complex

## Abstract

**Background:** Epilepsy is the most common neurological manifestation in individuals with tuberous sclerosis complex (TSC). However, real-world evidence on diagnosis and treatment patterns is limited. Here, we present data from TuberOus Sclerosis registry to increase disease Awareness (TOSCA) on changes in patterns of epilepsy diagnosis, treatments, and outcomes over time, and detailed epilepsy characteristics from the epilepsy substudy.

**Methods:** TuberOus Sclerosis registry to increase disease Awareness (TOSCA) was a multicentre, international disease registry, consisting of a main study that collected data on overall diagnostic characteristics and associated clinical features, and six substudies focusing on specific TSC manifestations. The epilepsy substudy investigated detailed epilepsy characteristics and their correlation to genotype and intelligence quotient (IQ).

**Results:** Epilepsy was reported in 85% of participants, more commonly in younger individuals (67.8% in 1970s to 91.8% in last decade), while rate of treatments was similar across ages (>93% for both infantile spasms and focal seizures, except prior to 1960). Vigabatrin (VGB) was the most commonly used antiepileptic drugs (AEDs). Individuals with infantile spasms showed a higher treatment response over time with lower usage of steroids. Individuals with focal seizures reported similar rates of drug resistance (32.5–43.3%). Use of vagus nerve stimulation (VNS), ketogenic diet, and surgery remained low.

**Discussion:** The epilepsy substudy included 162 individuals from nine countries. At epilepsy onset, most individuals with infantile spasms (73.2%) and focal seizures (74.5%) received monotherapies. Vigabatrin was first-line treatment in 45% of individuals with infantile spasms. Changes in initial AEDs were commonly reported due to inadequate efficacy. TSC1 mutations were associated with less severe epilepsy phenotypes and more individuals with normal IQ. In individuals with TSC diagnosis before seizure onset, electroencephalogram (EEG) was performed prior to seizures in only 12.5 and 25% of subsequent infantile spasms and focal seizures, respectively.

**Conclusions:** Our study confirms the high prevalence of epilepsy in TSC individuals and less severe phenotypes with *TSC1* mutations. Vigabatrin improved the outcome of infantile spasms and should be used as first-line treatment. There is, however, still a need for improving therapies in focal seizures. Electroencephalogram follow-up prior to seizure-onset should be promoted for all infants with TSC in order to facilitate preventive or early treatment.

## Introduction

Epilepsy is a common manifestation of tuberous sclerosis complex (TSC), affecting 80–90% of individuals (1, 2). It usually presents during the first year of life with infantile (epileptic) spasms or focal seizures. Focal seizures remain the most frequent type after the first year of life, but individuals with TSC may develop almost all seizure types. In about two-thirds of individuals with TSC, seizures are refractory to anticonvulsant treatment (3), a much higher proportion than the 23% reported in the general epilepsy population (4). Epilepsy is associated with a wide range of TSC-associated neuropsychiatric disorders (TAND) including intellectual disability (ID), autism spectrum disorder (ASD), and attention deficit hyperactivity disorder (ADHD), as well as impaired health-related quality of life (HRQoL) (5–9).

Treatment options for TSC-associated epilepsy in the first year of life are specific to infantile spasms because of high rates and individuals' responsiveness to vigabatrin (VGB), a first-line treatment option. Antiepileptic drug (AED) recommendations in TSC after the age of 1 year are the same as in the general epilepsy population based on seizure types. Candidates for epilepsy surgery should be identified early in the course of the disease. Other non-pharmacological treatment options including ketogenic diet, and vagus nerve stimulation (VNS) should also be considered early if the epilepsy is refractory (10). Evidence supports the use of mammalian target of rapamycin (mTOR)-inhibitors as adjunctive treatment to AEDs for treating focal epilepsy in TSC individuals, with a higher response rate in the younger subgroup aged below 6 years (11–13). Given the early onset, severity and significant impact of TSC-associated epilepsy on quality of life (QoL) (3, 5, 6), there is value in longitudinal population-based studies of detailed epilepsy characteristics.

The TuberOus Sclerosis registry to increase disease Awareness (TOSCA), which included individuals from 170 sites in 31 countries, was conceived to expand our understanding of different TSC manifestations, treatment patterns, and outcomes (14). TuberOus Sclerosis registry to increase disease Awareness consisted of a main study representing the diagnostic characteristics and associated clinical features, and six substudies, each focusing on specific TSC manifestations. In our initial publication, we reported characteristics of TSC-associated epilepsies (2). The key observations were (a) a typical onset pattern of focal seizures and infantile spasms in the first two years of life, (b), high rates of drug resistance in focal seizures compared to infantile spasms, and (c) a low proportion of individuals treated with non-pharmacological therapies, including epilepsy surgery. Here, we present data from the TOSCA final analysis, describing rates of epilepsy, treatment interventions, and outcomes over time. We also report findings from the epilepsy substudy, a TOSCA research project, aimed at reporting more detailed epilepsy characteristics including time to epilepsy diagnosis, electroencephalogram (EEG) patterns, and therapies.

## Methods

TuberOus Sclerosis registry to increase disease Awareness was a multicentre, international disease registry. The study methods have been reported in detail previously (14). In the main study, general background information (i.e., demographic data, family history, pre-natal history, and disease features such as neurological and neuropsychiatric, renal, cardiovascular, pulmonary, dermatological, and others) were collected retrospectively at baseline (first inclusion visit) followed by prospective data collection during an observation period of up to 5 years. Follow-up visits were scheduled according to the standard practice of the site and per the treating physician's best judgement, but at minimum intervals of 12 months. Data were retrieved from clinical records, electronic medical records, individuals' questionnaires, and *ad-hoc* clinical databases. Research projects were designed to record additional, more detailed data related to specific disease manifestations [i.e., subependymal giant cell astrocytoma (SEGA), renal angiomyolipoma, lymphangioleiomyomatosis, genetics, TAND, QoL, and epilepsy].

### Participants and Procedure

Individuals of any age who fulfilled clinical criteria for TSC diagnosis and a documented clinical visit for TSC within the past 12 months or newly diagnosed with TSC were enrolled in the main study. Investigators, specialized in epilepsy care, from 27 sites across nine countries (Belgium, France, Germany, Italy, Poland, Slovenia, Spain, Japan, and Turkey) participated in the epilepsy substudy.

Given the observational nature of the study, both diagnostic and treatment/management were performed according to local best practice. The study protocol, therefore, did not request any particular additional clinical or laboratory investigations.

Both main and substudy were designed and conducted according to the Guidelines for Good Clinical Practice and ethical principles outlined in the Declaration of Helsinki. Written informed consent was obtained from all individuals, parents, or guardians prior to enrolment, with prior endorsement by the local human research ethics committee.

### Data Analyses

From the main study, we present epilepsy characteristics, emphasizing changes in the rates of epilepsy diagnosis, treatment, and outcome over time. From the epilepsy substudy, we report characteristics [age of onset, frequency, tuber numbers, treatments and treatments outcomes, and intelligence quotient (IQ) level] of individuals with infantile spasms and focal seizures and correlated them to genotype. We report the impact of epilepsy characteristics and EEG foci on intellectual ability, date of EEG compared to the date of the seizure onset in individuals with focal seizures and infantile spasms with TSC diagnosis prior to seizure onset, number of AEDs used at epilepsy diagnosis, and the reasons for changes in the AED regimen. Intellectual ability was categorized as normal (IQ > 70), mild ID (IQ 51–70), moderate ID (IQ 36–50), severe ID (IQ 20–35), and profound ID (IQ <20). The response of individuals with infantile spasms was defined as follows: spasm-free + hypsarrhythmia resolved + normalized EEG or spasms free with disappearance of hypsarrhythmia, but persistent EEG anomalies. Efficacy in focal seizures was defined as >50% decrease in seizure frequency with rates of seizure freedom and response of >75%.

All eligible individuals enrolled in the TOSCA registry and epilepsy substudy, without any major protocol deviations, were included. As the study was observational in nature, primarily descriptive statistic methods were used. Continuous variables were evaluated quantitatively (frequency, mean, standard deviation, median, range), and categorical variables (presence/absence of a manifestation) were analyzed in terms of frequency distribution at baseline and at follow-up visits.

## Results

### Findings From the Final Analysis of the Main Study

#### Clinical Characteristics of Epilepsy

Of the 2,211 individuals enrolled in the TOSCA main study, 1,879 (85%) were reported to have epilepsy. Of these, 942 (50.1%) were female and 937 (49.9%) were male (Table 1). Focal seizures were reported in 1,343 individuals (71.5%), while infantile spasms were reported in 735 (39.1%). Five hundred and thirty-seven individuals (28.6%) were reported to have other seizure types. The median age at diagnosis was 1 year (range 0–66) for focal seizure and <1 year (range 0–30) for infantile spasms, respectively. Genetic data were available for 849 individuals with epilepsy; 587 (69.1%) had pathogenic mutations in the *TSC2* gene, while 155 (18.3%) had mutations in the *TSC1* gene. In 107 individuals (12.6%), no mutations were identified.

**Table 1:** Demographics and characteristics of individuals with TSC and epilepsy.

**Characteristics**	**Individuals**
**No. of individuals with epilepsy**, ***n*****(%)**	1,879 (85.0)
**Sex**, ***n*****(%)**	
Male	937 (49.9)
Female	942 (50.1)
**Age of individuals in years**, ***n*****(%)**	
≤ 2	257 (13.7)
>2 to ≤ 5	282 (15.0)
>5 to ≤ 9	311 (16.6)
>9 to ≤ 14	276 (14.7)
>14 to ≤ 18	126 (6.7)
>18 to ≤ 40	499 (26.6)
≥40	128 (6.8)
**Type of epilepsy**, ***n*****(%)**	
Infantile spasms	735 (39.1)
Focal seizures	1,343 (71.5)
Other seizures	537 (28.6)
**Median (range) age at diagnosis, years**	
Infantile spasms	<1 (0–30)
Focal seizures	1 (0–66)
**Genetic analysis**	
**Individuals with mutational analysis data available**, ***n*****(%)**	849 (45.2)
*TSC1*	155 (18.3)
*TSC2*	587 (69.1)
No mutation identified	107 (12.6)

*SD, standard deviation*.

#### Epilepsy Diagnosis, Treatment, and Outcome Patterns Over Time

**Figure 1:**
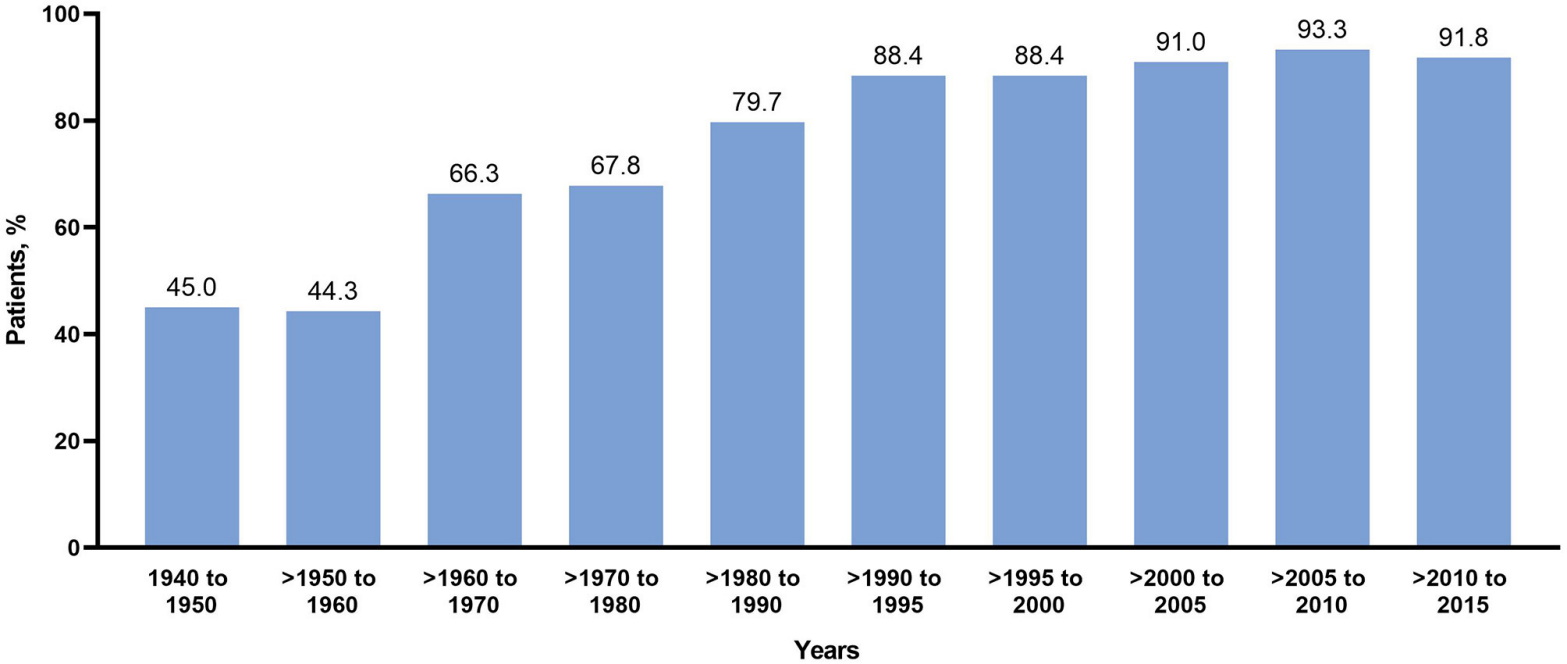
Rates of diagnosis of infantile spasms and focal seizures over time.

Epilepsy diagnosis was more common in younger individuals, ranging from 67.8% in the 1970s to 91.8% in the last decade (Figure 1, Table 2). The rates of infantile spasms diagnosis increased from 24.6% in the 1960s to about 41.4% from the 1990s. The rates of focal seizures diagnosis increased from 29.6% in the 1950s to about 84% in 2000s (Figure 1).

**Table 2:** Rates of epilepsy and treatments over time among individuals with TSC and epilepsy.

**Characteristics**	**1940 to 1950**	**>1950 to 1960**	**>1960 to 1970**	**>1970 to 1980**	**>1980 to 1990**	**>1990 to 1995**	**>1995 to 2000**	**>2000 to 2005**	**>2005 to 2010**	**>2010 to 2015**
	***N* = 20**	***N* = 61**	***N* = 104**	***N* = 183**	***N* = 265**	***N* = 172**	***N* = 241**	***N* = 323**	***N* = 461**	***N* = 380**
**Individuals ever had epilepsy**, ***n*****(%)**	9 (45.0)	27 (44.3)	69 (66.3)	124 (67.8)	212 (79.7)	152 (88.4)	213 (88.4)	294 (91.0)	430 (93.3)	349 (91.8)
**Type of epilepsy^a^**										
Infantile spasms	0	4 (14.8)	17 (24.6)	29 (23.4)	70 (33.0)	63 (41.4)	80 (37.6)	122 (41.5)	178 (41.4)	172 (49.3)
Focal seizures	5 (55.6)	8 (29.6)	27 (39.1)	57 (46.0)	135 (63.7)	93 (61.2)	168 (78.9)	246 (83.7)	361 (84.0)	243 (69.6)
Other seizures	4 (44.4)	19 (70.4)	42 (60.9)	66 (53.2)	79 (37.3)	64 (42.1)	52 (24.4)	54 (18.4)	84 (19.5)	73 (20.9)
**Infantile spasms**										
Individuals received treatment, *n* (%)	0	3 (75.0)	16 (94.1)	27 (93.1)	66 (94.3)	62 (98.4)	78 (97.5)	116 (95.1)	176 (98.9)	170 (98.8)
**Type of treatment**, ***n*****(%)^b^**	**–**									
VGB		1 (33.3)	6 (37.5)	11 (40.7)	45 (68.2)	39 (62.9)	65 (83.3)	100 (86.2)	155 (88.1)	155 (91.2)
ACTH	**–**	0	3 (18.8)	6 (22.2)	20 (30.3)	21 (33.9)	13 (16.7)	16 (13.8)	25 (14.2)	17 (10.0)
Ketogenic diet	**–**	0	1 (6.3)	0	1 (1.5)	0	2 (2.6)	10 (8.6)	7 (4.0)	13 (7.6)
Fructose derivatives	**–**	0	0	0	0	2 (3.2)	2 (2.6)	3 (2.6)	5 (2.8)	4 (2.4)
Vagus nerve stimulation	**–**	0	0	0	1 (1.5)	2 (3.2)	1 (1.3)	7 (6.0)	7 (4.0)	0
mTOR inhibitors	**–**	0	1 (6.3)	1 (3.7)	4 (6.1)	1 (1.6)	4 (5.1)	4 (3.4)	16 (9.1)	29 (17.1)
Surgery	**–**	0	0	0	1 (1.5)	0	0	9 (7.8)	14 (8.0)	12 (7.1)
Other	**–**	2 (66.7)	13 (81.3)	17 (63.0)	39 (59.1)	35 (56.5)	36 (46.2)	39 (33.6)	83 (47.2)	78 (45.9)
**Treatment outcome**, ***n*****(%)**										
Resolved spontaneously	**–**	0	1 (6.3)	0	2 (3.0)	7 (11.3)	0	2 (1.7)	9 (5.1)	1 (0.6)
Controlled	**–**	3 (100.0)	9 (56.3)	19 (70.4)	44 (66.7)	43 (69.4)	69 (88.5)	99 (85.3)	138 (78.4)	137 (80.6)
Not-controlled	**–**	0	5 (31.3)	3 (11.1)	14 (21.2)	8 (12.9)	7 (9.0)	12 (10.3)	26 (14.8)	31 (18.2)
Unknown	**–**	0	1 (6.3)	5 (18.5)	6 (9.1)	4 (6.5)	2 (2.6)	3 (2.6)	3 (1.7)	1 (0.6)
**Focal seizures**										
Individuals received treatment, *n* (%)	5 (100.0)	8 (100.0)	26 (96.3)	53 (93.0)	131 (97.0)	90 (96.8)	166 (98.8)	243 (98.8)	360 (99.7)	238 (97.9)
**Type of treatment ^b^**										
VGB	1 (20.0)	4 (50.0)	11 (42.3)	27 (50.9)	81 (61.8)	60 (66.7)	128 (77.1)	168 (69.1)	264 (73.3)	183 (76.9)
ACTH	0	0	0	1 (1.9)	4 (3.1)	2 (2.2)	3 (1.8)	9 (3.7)	13 (3.6)	9 (3.8)
Ketogenic diet	0	0	0	0	2 (1.5)	0	6 (3.6)	26 (10.7)	16 (4.4)	28 (11.8)
Fructose derivatives	0	0	4 (15.4)	4 (7.5)	8 (6.1)	7 (7.8)	7 (4.2)	15 (6.2)	19 (5.3)	19 (8.0)
Vagus nerve stimulation	0	0	0	2 (3.8)	8 (6.1)	10 (11.1)	8 (4.8)	19 (7.8)	13 (3.6)	3 (1.3)
mTOR inhibitors	0	0	1 (3.8)	9 (17.0)	17 (13.0)	10 (11.1)	24 (14.5)	40 (16.5)	37 (10.3)	43 (18.1)
Surgery	0	0	0	2 (3.8)	8 (6.1)	6 (6.7)	10 (6.0)	25 (10.3)	35 (9.7)	21 (8.8)
Other	5 (100.0)	5 (62.5)	23 (88.5)	39 (73.6)	96 (73.3)	61 (67.8)	116 (69.9)	179 (73.7)	244 (67.8)	166 (69.7)
**Treatment outcome**, ***n*****(%)**										
Resolved spontaneously	0	0	0	0	0	1 (1.1)	1 (0.6)	2 (0.8)	6 (1.7)	0
Controlled	3 (60.0)	5 (62.5)	18 (69.2)	30 (56.6)	75 (57.3)	50 (55.6)	106 (63.9)	139 (57.2)	212 (58.9)	134 (56.3)
Not-controlled	2 (40.0)	3 (37.5)	7 (26.9)	21 (39.6)	52 (39.7)	39 (43.3)	54 (32.5)	98 (40.3)	137 (38.1)	100 (42.0)
Unknown	0	0	1 (3.8)	2 (3.8)	4 (3.1)	0	5 (3.0)	4 (1.6)	5 (1.4)	4 (1.7)

*ACTH, adrenocorticotropic hormone; mTOR, mammalian target of rapamycin; VGB, vigabatrin*.

a *Individuals may have more than one type of epilepsy*.

b *Individuals may have received treatment as monotherapy or as combination therapy*.

More than 93% reported treatment for infantile spasms or focal seizures after 1960 (Table 2). Vigabatrin was the most commonly used AEDs in TSC individuals with infantile spasms and focal seizures in any year, with usage increasing over time and a clear shift after the late 1990s (>1950–1960: 33 and 50%; >1960–1970: 37.5 and 42.3%; >1970–1980: 40.7 and 50.9%; >1980–1990: 68.2 and 61.8%; >1990–1995: 62.9 and 66.7%, >1995–2000: 83.3 and 77.1%; >2000–2005: 86.2 and 69.1%; >2005–2010: 88.1 and 73.3%; >2010–2015: 91.2 and 76.9%). In contrast, usage of steroids for infantile spasms was at a peak (reported in 33.9%) between 1990 and 1995, decreasing thereafter (>1995–2000:16.7%; >2000–2005: 13.8%; >2005–2010: 14.2%; >2010–2015: 10.0%).

Epilepsy surgery for infantile spasms was reported in only one of 66 individuals (1.5%) between 1980 and 1990. Epilepsy surgery appeared as an alternative treatment for infantile spasms since 2000 and reached a peak in recent years (8.0% during 2005 and 2010). In individuals with focal seizures, use of the ketogenic diet was first reported in two individuals (1.5%) in 1980 with more regular use since 1995. Use of VNS was first reported in the late 1980s in patients with infantile spasms. Use of VNS showed a peak at the beginning of the 2000s (reported in 6% of individuals with infantile spasms and 7.8% in those with focal seizures), but there was a clear decrease thereafter. In contrast, the ketogenic diet showed a slow increase since its first use in this cohort but did not exceed 9% for infantile spasms and 12% for focal seizures. The use of mTOR inhibitors was reported in 17.1% of individuals with infantile spasms and 18.1% of individuals with focal seizures between 2010 and 2015.

**Figure 2:**
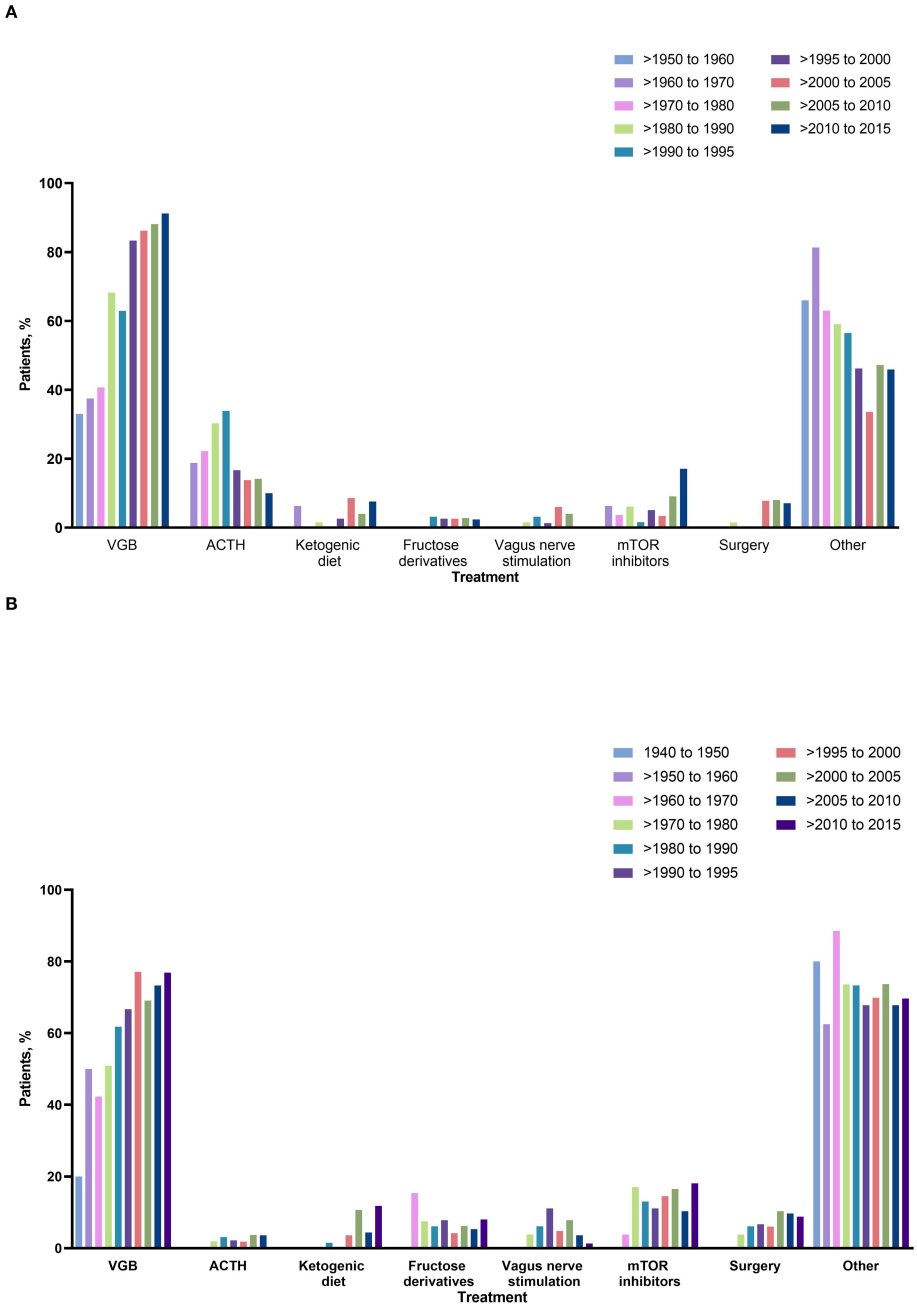
Types of intervention over time in individuals with **(A)** infantile spasms and **(B)** focal seizures. ACTH, adrenocorticotropic hormone; mTOR, mammalian target of rapamycin; VGB, vigabatrin.

Over time, individuals with infantile spasms responded better to treatment than those with focal seizures (Table 2); those with infantile spasms achieved a high response rate with a plateau since the late 1990s. This correlated to an increased use of VGB and a decreased use of steroids (Figure 2, Table 2). Outcome of focal seizures did not vary much since the 1960s, plateauing between 56 and 64% (Table 2).

### Findings From the Epilepsy Substudy

A total of 162 individuals (65 adults and 97 children) from 27 sites across nine countries were enrolled into the epilepsy substudy; 74 (45.7%) were males and 88 (54.3%) were females. The median age at enrolment was 14 years (range 2–63 years). Median duration of epilepsy prior to enrolment was 12 years (range 1–63 years).

Information about the type of treatment at epilepsy diagnosis was available in 68 of 71 individuals with infantile spasms and in 88 of 94 of those with focal seizures; 52 individuals (73.2%) with infantile spasms and 70 (74.5%) with focal seizures received monotherapies, while 16 (22.5%) with infantile spasms and 18 (19.1%) with focal seizures received polytherapies.

Changes of the initial antiepileptic treatment were reported in 64 (90.1%) individuals with infantile spasms and 64 (68.1%) with focal seizures. Most frequently reported reasons for change of treatment were partial or lack of efficacy of the first therapy. Vigabatrin was used as first-line therapy in individuals with infantile spasms in less than half of cases (45.1%) and was the most frequent second line treatment option (62.5%, Table 3).

**Table 3:** Initial and change in the treatment and reason for change in the epilepsy substudy.

	**Infantile spasms**	**Focal seizures**
**Number of individuals**	71 (43.8)	94 (58.0)
**Type of initial treatment reported at the epilepsy diagnosis**		
Monotherapy	52 (73.2)	70 (74.5)
VGB	32 (45.1)	33 (35.1)
ACTH	8 (11.3)	2 (2.1)
Other	12 (16.9)	33 (35.1)
Polytherapy	16 (22.5)	18 (19.1)
GABAergics and other	5 (7.0)	3 (3.2)
**Change of first treatment**	64 (90.1)	64 (68.1)
Median time from first to second treatment, days	214.0 (0–5,480)	288.0 (0–8,402)
**Type of second treatment**		
VGB	40 (62.5)	31 (48.4)
ACTH (steroids)	10 (15.6)	3 (4.7)
Ketogenic diet	1 (1.6)	0
Fructose derivates	1 (1.6)	1 (1.6)
Vagus nerve stimulation	0	0
mTOR inhibitors	0	0
Other	42 (65.6)	49 (76.6)
**Reason for change of drugs**	62 (96.9)	62 (96.9)
Partial efficacy	20 (31.3)	24 (37.5)
No efficacy	20 (31.3)	18 (28.1)
Side effects	1 (1.6)	4 (6.3)
Other	21 (32.8)	16 (25.0)

*ACTH, adrenocorticotropic hormone; mTOR, mammalian target of rapamycin; VGB, vigabatrin*.

*Values are expressed as n (%) unless otherwise mentioned*.

#### Characteristics of Epilepsy in TSC by Genotype

Of 63 individuals with available genetic data, 10 had pathogenic mutations in *TSC1* and 53 had pathogenic mutations in *TSC2*. Median age of epilepsy onset was 8 years in individuals with pathogenic variants in *TSC1* and <1 year in those with pathogenic variants in *TSC2*.

Infantile spasms were not reported in individuals with *TSC1*, but in 16 individuals (30.2%) with *TSC2*. Focal seizures were reported for most individuals (90% of *TSC1* individuals and 69.8% of *TSC2* individuals). The median frequency of focal seizures per week was 3.5 in individuals with pathogenic variants in *TSC1* and 1 in individuals with pathogenic variants in *TSC2*. MRI showed a mean number of three tubers in individuals with *TSC1* and 9.2 tubers in in *TSC2*.

Focal seizures were controlled with treatment in 60% of individuals with *TSC1* compared with only 22.6% of those with *TSC2*. Infantile spasms were controlled with treatment in 28.3% of individuals with *TSC2*.

#### Association of Epilepsy Foci With IQ Level

The association between epilepsy and IQ was examined in 102 individuals at baseline (69 had normal intellectual ability and 33 had various degrees of ID). Regarding IQ and focal spikes on last EEG recording, EEG showed temporal focal spikes in 52.3% of individuals with normal IQ and frontal focal spikes in 68% of individuals with moderate to severe ID.

#### Correlation of IQ Level and Genotype

Sixty-two of 102 individuals showed normal IQ level. The IQ level was normal in 70% of individuals with *TSC1* and in 20.8% of those with *TSC2*; moderate ID was found in 20% of individuals with *TSC1* and in 22.6% of those with *TSC2*; severe ID was observed in 15.1% of individuals with *TSC2*, but none of those with *TSC1*.

#### EEG in Individuals With TSC Diagnosis Before Seizures Onset

Diagnosis of TSC was established in 28 individuals before seizure onset. In this group, 16 individuals developed infantile spasms and 12 developed focal seizures. Median age at first EEG was 6 months in individuals with infantile spasms and 11 months in those with focal seizures. The first EEG was performed in 12.5% of individuals before the onset of infantile spasms and in 25% of individuals before the onset of focal seizures. Electroencephalogram was performed the same day seizures occurred in 18.8 and 16.7% of individuals with infantile spasms and focal seizures, respectively. In the remaining cases, 68.8 and 58.3%, EEG was performed after the onset of infantile spasms and focal seizures, respectively.

## Discussion

This study provides final data or information on epilepsy characteristics in a large cohort of TSC individuals who participated in the TOSCA registry and in the epilepsy substudy.

Findings from the main study emphasize the changes in both diagnosis and treatment patterns of TSC-associated epilepsies over time. Overall, a diagnosis of epilepsy was reported in approximately 85% of all individuals with TSC included, equally affecting both sexes. Infantile spasms were reported in about 39% of individuals with a median age of <1 year at diagnosis, and focal seizures in two-thirds of the individuals with a median age of 1 year at diagnosis. These findings were consistent with our previous report and also with other studies (2, 3, 15–17).

In our study, epilepsy diagnosis rates, especially diagnosis of infantile spasms, were higher in younger individuals (67.8% in 1970s to 91.8% in last decade). Since infantile spasms were reported as the seizures types of West syndrome by William West in 1841 (18), followed by Gibbs and Gibbs' description of the characteristic EEG pattern of hypsarrhythmia in 1952 (19), clinicians have made remarkable progress in recognizing this syndrome. The first proposal of classification of patients with epilepsies in syndromes published in the “Guide Bleu” (Blue Guide) in 1984 added to this knowledge (20). In addition, the better recognition of infantile spasms in TSC and their focal nature might have changed the delineation of focal seizures and infantile spasms in the recent years. Although we believe that there was an improvement in the diagnosis of infantile spasms and that this major improvement in clinical epileptology guarantees earlier and better seizure and developmental outcomes. We should be cautious about the concept of an increased rate of epilepsy diagnosis because older individuals in TSC clinics often have a lower rate of epilepsy as they present with angiomyolipoma or being the parent of a child with TSC.

Our data showed a better treatment response rate in individuals with infantile spasms over time, but not in those with focal seizures. This seems to be due to VGB specifity in infants with West syndrome and its growing usage since the 1990s. A decrease in the use of steroids after VGB also clearly shows the specific efficacy of VGB and the lack of a need to add steroids as practiced in infantile spasms due to other etiologies (21). Vigabatrin is an established first-line therapy for individuals with infantile spasms (10, 22). This precision medicine approach in individuals with infantile spasms in TSC is a major example of how an early diagnosis of TSC can help to better target the therapy and to avoid therapeutic failures and ineffective polytherapies. In addition, VGB is recommended as first-line treatment for focal seizures in individuals with TSC in the first year of life (10), aiming to prevent transition into infantile spasms. However, its use for focal seizures in older individuals does not seem to be superior to other AEDs licensed for focal seizures. Indeed, there has been no change in responder rates for focal seizures for the past 45 years despite availability of over 30 new AEDs (23, 24). This finding is also in line with the high percentage of drug resistance reported in individuals with TSC-associated focal seizures in recent reports (3, 25). Despite the high response to VGB in individuals with infantile spasms, it was not always the first therapy in individuals with infantile spasms (only 45% received VGB as first-line monotherapy). This finding is unexpected, especially in epilepsy centers, but emphasizes the need for more education about the use of individualized treatment options for specific etiologies.

Surprisingly, other non-pharmacological therapies such as VNS and the ketogenic diet were not used in this highly drug resistant population (range 1.5–8.6%). This might be due to the lack of randomized controlled trials in both therapies and evidence often based on retrospective small series (26) from one hand and the lack of expertise in both therapies on the other hand. The use of VNS in this cohort decreased during recent years after a peak in the 2000 and might be still underused although recommended as last resort in patients with refractory seizures.

Early evaluation for epilepsy surgery candidates in individuals with drug resistant TSC-associated epilepsies should be performed in expert centers in order to prevent/minimize developmental consequences of ongoing seizures (27). In our study, only a few individuals had epilepsy surgery. However, we did not ask in the study protocol how many had undergone pre-surgical evaluation.

Epilepsy surgery shows a relevant rate of 8–10% in our study but might not reflect yet the number of patients that were good candidates for epilepsy surgery and that can benefit from such therapy. Additionally, not all of the epilepsy centers participating in the study were also surgery centers trained in TSC-associated epilepsy. Therefore, additional training and education are needed and additional collaboration with expert surgery centers should be established for individuals with drug-resistant epilepsy with TSC in order to promote early identification of surgery good candidates.

Individuals with TSC and epilepsy are prescribed with multiple AEDs or undergo multiple surgical procedures to manage epileptic seizures (28, 29). However, we have observed in our epilepsy substudy that a large number of individuals were initiated on AED monotherapies as recommended by the ILAE (International League Against Epilesy). This might be related to the use of VGB in the first year of life in both infantile spasms and focal seizures or epilepsy combining both seizure types.

Our results also showed the increased use of disease-modifying treatment with mTOR inhibitors. The efficacy of this therapy was reported in late 2010 and its use in case of failure of initial treatment could be the rational approach. Its use increased and reached 18% in the last reports from the TOSCA study in 2015, showing the need for more efficient therapies in focal seizures associated with TSC. This increased use of approved mTOR inhibitor, everolimus, and the wider evaluation of surgery candidates in the management of TSC-associated focal seizures and in some individuals with drug-resistant infantile spasms might improve responder rates in the future and could help to achieve a better cognitive TAND outcomes.

In our substudy, in infants with TSC diagnosis prior to seizure onset, EEG was performed mainly after the onset of clinical seizures, both for infantile spasms (in 68.8%) and focal seizures (in 58.3%). Curatolo et al. recommended in 2012 (22) and later in 2018 (10) to use EEG in infants with TSC before seizure onset to early identify individuals at high risk of developing epilepsy. This was also reported in the international recommendations (guidelines) in 2013 (30), based on studies showing that TSC individuals who were diagnosed and treated before the onset of seizures had less severe epilepsy and better neurodevelopmental outcomes (31). Abnormal EEG patterns and/or in some instances subclinical seizures recorded on the EEG should urge the use of AED therapy without waiting for the onset of overt clinical seizures. The results of the research project are in contrast with these recommendations and emphasize the need for more information for clinicians about the key role of sequential EEG recordings to early recognize individuals at high risk of developing early onset seizures and preventive AED treatment. Parents should be educated to recognize seizures earlier and most importantly EEG recordings should be performed—with an ultrasound of cardiac rhabdomyoma, pre-natal, or post-natal MRIs—in cases with family history of TSC with signs of TSC or with cutaneous hallmarks of TSC.

The place of this pre-symptomatic diagnosis strategy for epilepsy in TSC and the preventive therapy might be better implemented after the recent validation of this approach with the first results of the EPISTOP study (32, 33). Individuals receiving early preventive treatment showed a later epilepsy onset and a less severe epilepsy compared to those receiving standard therapy started after the onset of clinical seizures. The cognitive outcome might need further validation and longer follow-up (32, 33).

Our data show no significant correlation between the spikes focus and the IQ levels as for frontal or temporal focus. More severe cognitive but mainly psychiatric disorders as ASD are reported with temporal lesions (34). However, we did not report ASD testing and TAND results were mostly missing.

Finally, our study showed that individuals with *TSC1* had less severe phenotypes than those with *TSC2*. This finding is in accordance with the literature (3, 25, 35), but, importantly, we were able to validate it on a very large cohort probably less biased than mono-center studies and smaller series. A higher proportion of individuals with *TSC1* had normal IQ levels than those with *TSC2*. Compared to individuals with *TSC2*, they had fewer numbers of tubers, later onset of epilepsy, and higher rates of controlled seizures. The tuber load, usually higher in individuals with *TSC2*, might have a role in creating more complex and diffuse abnormal networks, with fewer regions showing normal brain cortex, leading more frequently to drug resistant epilepsy and higher rates of co-morbidities.

In conclusion, our study highlights that despite the improvement in diagnosis and in some aspects of treatment of TSC-associated epilepsy over time, especially for infantile spasms, there are still some major improvements to be made. Better epilepsy control is urgently needed, mainly for focal seizures. A more targeted use of available therapies and the promotion of innovative therapies and of evaluating surgery candidates should continue. Despite the established guidelines, the need for further education of clinicians in order to provide earlier diagnosis of epilepsy based on serial EEGs before the onset of seizures in patients with TSC should be promoted and to use VGB as first monotherapy for infantile spasms established as the first line therapy. Pre-seizure diagnosis will also help to use timely or even preventive therapies and could be a major step toward changing the natural history of epilepsy in individuals with TSC. Finally, the use of new targeted therapies such as mTOR inhibitors, or cannabidiol (36), and earlier and better definition of candidates for epilepsy surgery may lead to better outcomes, especially for focal seizures where the seizure control rates have plateaued in the last decade.

## TOSCA Investigators

Japan: Nobuo Shinohara, Shigeo Horie, Masaya Kubota, Jun Tohyama, Katsumi Imai, Mari Kaneda, Hideo Kaneko, Yasushi Uchida, Tomoko Kirino, Shoichi Endo, Yoshikazu Inoue, Katsuhisa Uruno; Turkey: Ayse Serdaroglu, Zuhal Yapici, Banu Anlar, Sakir Altunbasak; Russia: Olga Lvova, Oleg Valeryevich Belyaev, Oleg Agranovich, Elena Vladislavovna Levitina, Yulia Vladimirovna Maksimova, Antonina Karas; China: Yuwu Jiang, Liping Zou, Kaifeng Xu, Yushi Zhang, Guoming Luan, Yuqin Zhang, Yi Wang, Meiling Jin, Dingwei Ye, Weiping Liao, Liemin Zhou, Jie Liu, Jianxiang Liao, Bo YAN, Yanchun Deng, Li Jiang, Zhisheng Liu, Shaoping Huang, Hua Li; Korea: Kijoong Kim; Taiwan: Pei-Lung Chen, Hsiu-Fen Lee, Jeng-Dau Tsai, Ching-Shiang Chi, Chao-Ching Huang; Australia: Kate Riney, Deborah Yates, Patrick Kwan; Thailand: Surachai Likasitwattanakul, Charcrin Nabangchang, Lunliya Thampratankul Krisnachai Chomtho, Kamornwan Katanyuwong, Somjit Sriudomkajorn; South Africa: Jo Wilmshurst; Israel: Reeval Segel, Tal Gilboa, Michal Tzadok, Aviva Fattal- Valevski; Greece: Panagiotis Papathanasopoulos, Antigone Syrigou Papavasiliou, Stylianos Giannakodimos, Stylianos Gatzonis, Evangelos Pavlou, Meropi Tzoufi; Netherlands: A.M.H. Vergeer; Belgium: Marc Dhooghe, Hélène Verhelst, Filip Roelens, Marie Cecile Nassogne, Pierre Defresne, Liesbeth De Waele, Patricia Leroy, Nathalie Demonceau, Benjamin Legros, Patrick Van Bogaert, Berten Ceulemans, Lina Dom; France: Pierre Castelnau, Anne De Saint Martin, Audrey Riquet, Mathieu Milh, Claude Cances, Jean-Michel Pedespan, Dorothee Ville, Agathe Roubertie, Stéphane Auvin, Patrick Berquin, Christian Richelme, Catherine Allaire, Sophie Gueden, Sylvie Nguyen The Tich, Bertrand Godet; Spain: Maria Luz Ruiz Falco Rojas, Jaume Campistol Planas, Antonio Martinez Bermejo, Patricia Smeyers Dura, Susana Roldan Aparicio, Maria Jesus Martinez Gonzalez, Javier Lopez Pison, Manuel Oscar Blanco Barca, Eduardo Lopez Laso, Olga Alonso Luengo, Francisco Javier Aguirre Rodriguez, Ignacio Malaga Dieguez, Ana Camacho Salas, Itxaso Marti Carrera, Eduardo Martinez Salcedo, Maria Eugenia Yoldi Petri, Ramon Cancho Candela; Portugal: Ines da Conceicao Carrilho, Jose Pedro Vieira, José Paulo da Silva Oliveira Monteiro, Miguel Jorge Santos de Oliveira Ferreira Leao, Catarina Sofia Marceano Ribeiro Luis, Carla Pires Mendonca; Lithuania: Milda Endziniene; Latvia: Jurgis Strautmanis; Estonia: Valentin Sander, Inga Talvik; Italy: Maria Paola Canevini, Antonio Gambardella, Dario Pruna, Salvatore Buono, Elena Fontana, Bernardo Dalla Bernardina; Romania: Carmen Burloiu, Iuliu Stefan Bacos Cosma, Mihaela Adela Vintan, Laura Popescu; Czech Republic: Karel Zitterbart; Slovakia: Jaroslava Payerova, Ladislav Bratsky, Zuzana Zilinska; Austria: Ursula Gruber-Sedlmayr, Matthias Baumann, Edda Haberlandt, Kevin Rostasy, Ekaterina Pataraia; United Kingdom: Frances Elmslie, Clare Ann Johnston, Pamela Crawford; Denmark: Peter Uldall; Sweden: Paul Uvebrant, Olof Rask; Norway: Marit Bjoernvold, Eylert Brodtkorb, Andreas Sloerdahl, Ragnar Solhoff, Martine Sofie Gilje Jaatun; Poland: Marek Mandera, Elzbieta Janina Radzikowska, Mariusz Wysocki; Germany: Michael Fischereder, Gerhard Kurlemann, Bernd Wilken, Adelheid Wiemer-Kruel, Klemens Budde, Klaus Marquard, Markus Knuf, Andreas Hahn, Hans Hartmann, Andreas Merkenschlager, Regina Trollmann.

## Data Availability Statement

Novartis supports the publication of scientifically rigorous analysis that is relevant to patient care, regardless of a positive or negative outcome. Qualified external researchers can request access to anonymized patient-level data, respecting patient informed consent, contacting study sponsor authors. The protocol can be accessed through EnCePP portal http://www.encepp.eu/ (EU PAS Register Number EUPAS3247).

## Ethics Statement

The study protocol and all amendments were reviewed and approved (if applicable) by independent ethics committee/institutional review board for each center: National Hospital Organization Central Ethics Committee; Gazi University Clinical Research Ethics Committee; Independent Multidisciplinary Committee on Ethical Review of Clinical Trials; Peking Union Medical College Hospital; Commissie Medische Ethiek UZ Brussel; CNIL (Commission National de l'Informatique et des Libertés), CCTIRS (Comité Consultatif sur le Traitement de L'Information en matière de Recherche dans le domaine de la Santé); Comité Etico Investigación Clínica de Euskadi (CEIC-E); Consejeria de Salud y Bienestar Social, Dirección General de Calidad, Investigación, Desarrollo e Innovación, Comité Coordinador de Ética de la Investigación Biomédica de Andalucía; Research Ethics Committee of the University of Tartu (UT REC); Ethikkommission der Medizinischen Universität Graz; North Wales REC—West; Regionala Etikprövningsnämnden i Göteborg; REK—Regionale Komiteer for Medisinsk og Helsefaglig Forskningsetikk; Komisja Bioetyczna przy Instytucie Pomnik Centrum Zdrowia Dziecka; Ethikkommission bei der Ludwig-Maximilians-Universitat München; Hokkaido University Hospital Independent Clinical Research Institutional Ethics Committee; Medical Juntendo University Institutional Ethics Committee; National Center for Chile Health and Deveropment of IRB; Osaka University Hospital of IRB; Ethics Committee at Moscow Institute of Pediatrics and Pediatric Surgery; Peking University First Hospital; Sanbo Brain Hospital Capital Medical University; Tianjin Children's Hospital; Childrens Hospital of Fudan University; Zhongshan Hospital Fudan University; Fudan University Shanghai Cancer Center; The Second Affiliated Hospital of Guangzhou Medical University; The First Affiliated Hospital, Sun Yan-Sen University; The First Affiliated Hospital Of Guangzhou Medical University; Shenzhen Children's Hospital; West China Hospital, Sichuan University; Xijing Hospital; Children's Hospital of Chongqing Medical University; Wuhan Children's Hospital; The Second Affiliated Hospital of Xi'an Jiaotong University; Guangdong 999 Brain Hospital; Seoul National University Hospital Institutional Review Board; National Taiwan University Hospital (NTUH) Research Ethics Committee (REC); Institutional Review Board of the Taichung Veterans General Hospital; Institutional Review Board of Chung Shan Medical University Hospital; Institutional Review Board, Tungs' Taichung MetroHarbor Hospital; Institutional Review Board of National Cheng Kung University Hospital; Metro South Human Research Ethics Committee; Sydney Children's Hospital Network Human Research Ethics Committee; St Vincents Hospital Human Research Ethics Committee; Royal Melbourne Hospital Human Research Ethics Committee; Siriraj Institutional Review Board; The Institutional Review board, Faculty of Medicine, Chulalongkorn University, 3rd Floor, Ananthamahidol Building, King Chulalongkorn Memorial Hospital; The Committee on Human Rights Related to Research Involving Human Subjects; Institutional Review board, Royal Thai Army Medical Department IRB RTA, 5th Floor, Phramongkutklaowejvitya Building, Phramongkutklao College of Medicine; Research Ethics Committee, Faculty of Medicine, Chiang Mai University; Research and Development, Queen Sirikit National Institute of Child Health; Human Research Ethics Committee, Faculty of Health Sciences, University of Cape Town; Shaare Zedek Meidcla Center Helsinki Comittee; Sheba Medical Center Helsinki Comittee; Tel Aviv Sourasly Medical Center Helsinki Comittee; General University Hospital of Patras Ethics Committee; Pendeli Children's Hospital Ethics Committee; General University Hospital of Athens 'G. Gennimatas Ethics Committee; Evaggelismos General Hospital Ethics Committee; General University Hospital of Thessaloniki AHEPA Ethics Committee; General University Hospital of Ionnina Ethics Committee; METC UMC Utrecht; Direcció General de Regulació, Planificació i Recursos Sanitaris; Comité Ético de Investigación Clínica del Hospital Universitario Vall d'Hebron de Barcelona, Generalitat de Catalunya. Departament de Salut; Comité Ético de Investigación Clínica Hospital Universitario La Paz; Dirección General de Ordenación e Inspección, Consejería de Sanidad Comunidad de Madrid, Servicios de Control Farmacéutico y Productos Sanitarios; Comité Etico Investigación Clínica del Hospital Universitario y Politécnico de La Fe; Dirección General de Farmàcia i Productes Sanitaris, Generalitat de Valencia; Comité de Ética de la Investigación de Centro de Granada; Instituto Aragonés de Ciencias de la Salud (IACS); Comité Etico Investigación Clínica Regional del Principado de Asturias; Comité Etico Investigación Clínica Hospital 12 de Octubre; Comité Etico Investigación Clínica Hospital Universitario Virgen de la Arrixaca; Sección de Ordenación e Inspección Farmacéutica Departamento de Salud; Comité Ético de Investigación Clínica del Hospital Universitario del Río Hortega de Valladolid; Comissão de Ética para a Saúde (CES), Centro Hospitalar de Lisboa Ocidental, EPE; Comissão de Ética para a Saúde (CES), Centro Hospitalar do Porto, E.P.E; Comissão de Ética para a Saúde (CES), Centro Hospitalar Lisboa Central, EPE; Comissão de Ética para a Saúde (CES), Hospital Garcia de Orta, EPE; Comissão de Ética para a Saúde (CES), Centro Hospitalar de São João, EPE; Comissão de Ética para a Saúde (CES), Hospital Professor Doutor Fernando Fonseca, EPE; Comissão de Ética para a Saúde (CES), Centro Hospitalar do Algarve, EPE (Unidade de Faro); LUHS Kaunas Regional Biomedical Research Ethics Committee; Paula Stradina kliniskās universitātes slimnicas, Attistibas biedribas Kliniskās izpētes Etikas komiteja, Ethics Committee for Clinical Research; Komisija Republike Slovenije za medicinsko etiko; Comitato Etico Indipendente Presso La Fondazione Ptv Policlinico Tor Vergata Di Roma; Comitato Etico Regione Calabria Sezione Centro c/o A.O.U. Mater Domini Di Catanzaro; Comitato Etico Azienda Ospedaliera Universitaria Di Cagliari; Comitato Etico Cardarelli-Santobono c/o Ao Cardarelli; Comitato Etico Per La Sperimentazione Clinica Delle Province Di Verona E Rovigo, Presso Aoui Verona; Eticka Komise Fn Brno; Eticka Komisia Dfnsp Bratislava; Eticka Komisia Pri Dfn Kosice; Eticka Komisia Bratislavskeho Samospravneho Kraja; Comisia Naţională de Bioetică a Medicamentului i a Dispozitivelor Medicale; Comitato Etico Milano area 1 c/o ASST FBF Sacco - P.O. L. Sacco; Comité de Ética de la Investigación de Centro Hospital Universitario Virgen del Rocío; Comité Ético de Investigación Clínica Fundació Sant Joan de Déu Generalitat de Catalunya. Departament de Salut; Comité Ético de Investigación Clínica Hospital Infantil Universitario Niño Jesús; Consejería de Sanidad Dirección General de Salus Pública Junta de Castilla León; Dirección General de Asistencia Sanitaria, Consejería de Sanidad Gobierno del Principado de Asturias; Dirección General de Planificación, Ordenación Sanitaria y Farmacéutica e Investigación, Consejeria de Sanidad y Política Social Región de Murcia; Ethics Committee at Moscow Institute of Pediatrics and Pediatric Surgery; Paula Stradina kliniskās Universitātes Slimnicas, Attistibas Biedribas Kliniskās Izpētes Etikas komiteja, Ethics Committee for Clinical Research; The First Affiliated Hospital of The Fourth Military Medical University; Zhongshan Hospital Fudan University. Written informed consent to participate in this study was provided by the participants' legal guardian/next of kin.

## Author Contributions

RN, EB, MB, PC, JF, MF, CH, SJ, JK, JL, AM, MS, RT, BZ, and AJ: designing the study, patient accrual, clinical care, data interpretation, drafting, revising, final review, and approval of the manuscript. PdV, CF, GB, TC, VC, FO'C, JQ, YT, and SY: designing the study, data interpretation, drafting, revising, final review, and approval of the manuscript. LD'A: designing the study, trial management, data collection, data analysis, data interpretation, drafting, revising, final review, and approval of the manuscript. RM: designing the study, data analysis, data interpretation, drafting, revising, final review, and approval of the manuscript. SS: designing the study, trial statistician, data analysis, data interpretation, drafting, revising, final review, and approval of the manuscript. All authors contributed to the article and approved the submitted version.

## Funding

The study was funded by Novartis Pharma AG. Novartis has contributed to study design, data analysis and the decision to publish. Novartis authors reviewed the draft for submission.

## Conflict of Interest

RN, EB, TC, VC, PC, GB, PdV, JK, JF, MF, CF, CH, SJ, FO'C, JQ, MS, RT, MD, JL, AM, SY, MB, BZ, and AJ received honoraria and support for the travels from Novartis. VC received personal fees for consulting, lecture fees and travel from Actelion, Bayer, Biogen Idec, Boehringer Ingelheim, Gilead, GSK, MSD, Novartis, Pfizer, Roche, Sanofi; grants from Actelion, Boehringer Ingelheim, GSK, Pfizer, Roche; personal fees for developing educational material from Boehringer Ingelheim and Roche. PdV has been on the study steering group of the EXIST-1, 2 and 3 studies sponsored by Novartis, and co-PI on two investigator-initiated studies part-funded by Novartis. RN received grant support, paid to her institution, from Eisai and lectures fees from Nutricia, Eisai, Advicenne, and GW Pharma. YT received personal fee from Novartis for lecture and for copyright of referential figures from the journals, and received grant from Japanese government for intractable epilepsy research. SJ was partly financed by the EC Seventh Framework Programme FP7/2007-2013; EPISTOP, grant agreement no. 602391, the Polish Ministerial funds for science years 2013-2018 for the implementation of international cofinanced project and the grant EPIMARKER of the Polish National Center for Research and Development No STRATEGMED3/306306/4/2016. JK, PC, CH, JL, and JQ received research grant from Novartis. RM and SS are employees of Novartis. LD'A was employee of Novartis at the time of manuscript concept approval. The authors declare that this study received funding from Novartis Pharma AG. The funder had the following involvement in the study: contribution to study design, data analysis and the decision to publish. Novartis authors reviewed the draft for submission.

## Publisher's Note

All claims expressed in this article are solely those of the authors and do not necessarily represent those of their affiliated organizations, or those of the publisher, the editors and the reviewers. Any product that may be evaluated in this article, or claim that may be made by its manufacturer, is not guaranteed or endorsed by the publisher.

## References

[1] Holmes GL Stafstrom CE. Tuberous sclerosis complex and epilepsy: recent developments and future challenges. Epilepsia. (2007) 48:617–30. 10.1111/j.1528-1167.2007.01035.x 17386056

[2] Nabbout R Belousova E Benedik MP Carter T Cottin V Curatolo P. Epilepsy in tuberous sclerosis complex: findings from the TOSCA study. Epilepsia Open. (2019) 4:73–84. 10.1002/epi4.12286 30868117 PMC6398114

[3] Chu-Shore CJ Major P Camposano S Muzykewicz D Thiele EA. The natural history of epilepsy in tuberous sclerosis complex. Epilepsia. (2010) 51:1236–41. 10.1111/j.1528-1167.2009.02474.x 20041940 PMC3065368

[4] Berg AT Vickrey BG Testa FM Levy SR Shinnar S DiMario F et al. How long does it take for epilepsy to become intractable? A prospective investigation. Ann Neurol. (2006) 60:73–9. 10.1002/ana.20852 16685695

[5] Vergeer M de Ranitz-Greven WL Neary MP Ionescu-Ittu R Emond B Sheng Duh M. Epilepsy, impaired functioning, and quality of life in patients with tuberous sclerosis complex. Epilepsia Open. (2019) 4:581–92. 10.1002/epi4.12365 31819914 PMC6885664

[6] Shepherd C Koepp M Myland M Patel K Miglio C Siva V. Understanding the health economic burden of patients with tuberous sclerosis complex (TSC) with epilepsy: a retrospective cohort study in the UK Clinical Practice Research Datalink (CPRD). BMJ Open. (2017) 7:e015236. 10.1136/bmjopen-2016-015236 28982809 PMC5640029

[7] Bar C Ghobeira R Azzi R Ville D Riquet A Touraine R. Experience of follow-up, quality of life, and transition from pediatric to adult healthcare of patients with tuberous sclerosis complex. Epilepsy Behav. (2019) 96:23–7. 10.1016/j.yebeh.2019.04.027 31077938

[8] Marques R Belousova E Benedik MP Carter T Cottin V Curatolo P. Treatment patterns and use of resources in patients with tuberous sclerosis complex: insights from the TOSCA registry. Front Neurol. (2019) 10:1144. 10.3389/fneur.2019.01144 31708865 PMC6823684

[9] Jansen AC Vanclooster S de Vries PJ Fladrowski C Beaure d'Augères G Carter T. Burden of illness and quality of life in tuberous sclerosis complex: findings from the TOSCA study. Front Neurol. (2020) 11:904. 10.3389/fneur.2020.00904 32982929 PMC7485558

[10] Curatolo P Nabbout R Lagae L Aronica E Ferreira JC Feucht M. Management of epilepsy associated with tuberous sclerosis complex: updated clinical recommendations. Eur J Paediatr Neurol. (2018) 22:738–48. 10.1016/j.ejpn.2018.05.006 29880258

[11] French JA Lawson JA Yapici Z Ikeda H Polster T Nabbout R. Adjunctive everolimus therapy for treatment-resistant focal-onset seizures associated with tuberous sclerosis (EXIST-3): a phase 3, randomised, double-blind, placebo-controlled study. Lancet. (2016) 388:2153–63. 10.1016/s0140-6736(16)31419-2 27613521

[12] Curatolo P Moavero R van Scheppingen J Aronica E. mTOR dysregulation and tuberous sclerosis-related epilepsy. Expert Rev Neurother. (2018) 18:185–201. 10.1080/14737175.2018.1428562 29338461

[13] Curatolo P Franz DN Lawson JA Yapici Z Ikeda H Polster T. Adjunctive everolimus for children and adolescents with treatment-refractory seizures associated with tuberous sclerosis complex: *post-hoc* analysis of the phase 3 EXIST-3 trial. Lancet Child Adolesc Health. (2018) 2:495–504. 10.1016/s2352-4642(18)30099-3 30169322

[14] Kingswood JC Bruzzi P Curatolo P de Vries PJ Fladrowski C Hertzberg C. TOSCA - first international registry to address knowledge gaps in the natural history and management of tuberous sclerosis complex. Orphanet J Rare Dis. (2014) 9:182. 10.1186/s13023-014-0182-9 25424195 PMC4256743

[15] Kingswood JC d'Augeres B Belousova E Ferreira JC Carter T Castellana R. TuberOus SClerosis registry to increase disease Awareness (TOSCA) - baseline data on 2093 patients. Orphanet J Rare Dis. (2017) 12:2. 10.1186/s13023-016-0553-5 28057044 PMC5217262

[16] Jeong A Wong M. Systemic disease manifestations associated with epilepsy in tuberous sclerosis complex. Epilepsia. (2016) 57:1443–9. 10.1111/epi.13467 27417921

[17] Dabora SL Jozwiak S Franz DN Roberts PS Nieto A Chung J. Mutational analysis in a cohort of 224 tuberous sclerosis patients indicates increased severity of TSC2, compared with TSC1, disease in multiple organs. Am J Hum Genet. (2001) 68:64–80. 10.1086/316951 11112665 PMC1234935

[18] West W. On a peculiar form of infantile convulsions. Lancet. (1841) 35:724–5. 10.1016/S0140-6736(00)40184-4

[19] Gibbs FA Gibbs EL. Atlas of Electroencephalography. Cambridge, MA: Addison-Wesley (1952).

[20] Proposal for revised classification of epilepsies and epileptic syndromes. Commission on Classification and Terminology of the International League Against Epilepsy. Epilepsia. (1989) 30:389–99. 10.1111/j.1528-1157.1989.tb05316.x 2502382

[21] O'Callaghan FJ Lux AL Darke K Edwards SW Hancock E Johnson AL. The effect of lead time to treatment and of age of onset on developmental outcome at 4 years in infantile spasms: evidence from the United Kingdom Infantile Spasms Study. Epilepsia. (2011) 52:1359–64. 10.1111/j.1528-1167.2011.03127.x 21668442

[22] Curatolo P Józwiak S Nabbout R. Management of epilepsy associated with tuberous sclerosis complex (TSC): clinical recommendations. Eur J Paediatr Neurol. (2012) 16:582–6. 10.1016/j.ejpn.2012.05.004 22695035

[23] Schulze-Bonhage A. A 2017 review of pharmacotherapy for treating focal epilepsy: where are we now and how will treatment develop? Expert Opin Pharmacother. (2017) 18:1845–53. 10.1080/14656566.2017.1391788 29140112

[24] Stevens CE Stafstrom CE. Pharmacotherapy for focal seizures in children and adolescents. Drugs. (2018) 78:1321–37. 10.1007/s40265-018-0959-6 30128698

[25] Jeong A Nakagawa JA Wong M. Predictors of drug-resistant epilepsy in tuberous sclerosis complex. J Child Neurol. (2017) 32:1092–98. 10.1177/0883073817737446 29129154 PMC5773119

[26] Elliott RE Rodgers SD Bassani L Morsi A Geller EB Carlson C. Vagus nerve stimulation for children with treatment-resistant epilepsy: a consecutive series of 141 cases. J Neurosurg Pediatr. (2011) 7:491–500. 10.3171/2011.2.peds10505 21529189

[27] Guerreiro MM Andermann F Andermann E Palmini A Hwang P Hoffman HJ. Surgical treatment of epilepsy in tuberous sclerosis: strategies and results in 18 patients. Neurology. (1998) 51:1263–9. 10.1212/wnl.51.5.1263 9818843

[28] Hsieh DT Jennesson MM Thiele EA. Epileptic spasms in tuberous sclerosis complex. Epilepsy Res. (2013) 106:200–10. 10.1016/j.eplepsyres.2013.05.003 23796861

[29] Song J Swallow E Said Q Peeples M Meiselbach M Signorovitch J. Epilepsy treatment patterns among patients with tuberous sclerosis complex. J Neurol Sci. (2018) 391:104–8. 10.1016/j.jns.2018.06.011 30103955

[30] Krueger DA Northrup H. Tuberous sclerosis complex surveillance and management: recommendations of the 2012 International Tuberous Sclerosis Complex Consensus Conference. Pediatr Neurol. (2013) 49:255–65. 10.1016/j.pediatrneurol.2013.08.002 24053983 PMC4058297

[31] Jozwiak S Słowińska M Borkowska J Sadowski K Łojszczyk B Domańska-Pakieła D. Preventive antiepileptic treatment in tuberous sclerosis complex: a long-term, prospective trial. Pediatr Neurol. (2019) 101:18–25. 10.1016/j.pediatrneurol.2019.07.008 31481332

[32] Moavero R Benvenuto A Emberti Gialloreti L Siracusano M Kotulska K. Early clinical predictors of autism spectrum disorder in infants with tuberous sclerosis complex: results from the EPISTOP study. J Clin Med. (2019) 8:788. 10.3390/jcm8060788 31163675 PMC6617179

[33] Kotulska K Kwiatkowski DJ Curatolo P Weschke B Riney K Jansen F. Prevention of epilepsy in infants with tuberous sclerosis complex in the EPISTOP Trial. Ann Neurol. (2021) 89:304–14. 10.1002/ana.25956 33180985 PMC7898885

[34] Bolton PF Park RJ Higgins JN Griffiths PD Pickles A. Neuro-epileptic determinants of autism spectrum disorders in tuberous sclerosis complex. Brain. (2002) 125:1247–55. 10.1093/brain/awf124 12023313

[35] Ogórek B Hamieh L Hulshof HM Lasseter K Klonowska K Kuijf H. TSC2 pathogenic variants are predictive of severe clinical manifestations in TSC infants: results of the EPISTOP study. Genet Med. (2020) 22:1489–97. 10.1038/s41436-020-0823-4 32461669

[36] Thiele EA Marsh ED French JA Mazurkiewicz-Beldzinska M Benbadis SR Joshi C. Cannabidiol in patients with seizures associated with Lennox-Gastaut syndrome (GWPCARE4): a randomised, double-blind, placebo-controlled phase 3 trial. Lancet. (2018) 391:1085–96. 10.1016/s0140-6736(18)30136-3 29395273

